# Self-Perceived Oral Symptoms Associated with Nicotine Replacement Therapy

**DOI:** 10.3290/j.ohpd.a45079

**Published:** 2020-09-04

**Authors:** Jane Manakil, Andrew Miliankos, Megan Gray, Roy George

**Affiliations:** a Senior Lecturer, School of Dentistry and Oral Health, Griffith University, Gold Coast Campus, Australia. Helped with planning the study design, questionnaire and statistics; initial write-up.; b General Dentist, Victoria, Australia. Survey distribution and management of questionnaire; collection of survey and data uploading; data analysis.; c Lecturer, Coordinator, Clinical Outplacements, DOH Contact Officer, School of Dentistry and Oral Health, Griffith University, Gold Coast Campus, Australia. Data processing; writing the manuscript.; d Discipline Head Endodontics, School of Dentistry and Oral Health, Griffith University, Gold Coast, Australia. Planning data presentation; reassigning the data analysis; proofreading the manuscript.

**Keywords:** nicotine replacement products, nicotine replacement therapy, oral health symptoms, tobacco

## Abstract

**Purpose:**

This study aimed to evaluate the experience of specific oral and dental symptoms or side effects as reported by patients following the use of nicotine replacement therapy (NRT) products.

**Materials and Methods:**

The study involved paper-based confidential survey questionnaires accessible for a period of 8 months to patients attending the School of Dentistry Dental Clinic, Griffith University, Australia. This study recorded demography, smoking history, NRT use history, and specific oral and systemic symptoms. The data was assessed and grouped into three divisions: those with no history of NRT use, current and former users of NRT, and current users of NRT.

**Results:**

Current users of NRT reported a statistically significantly higher incidence of all oral symptoms and increased incidence of systemic symptoms, as compared to those with no history of NRT use. There was no statistically significant difference between current and former users of NRT for almost all symptoms.

**Conclusions:**

A correlative relationship has been observed between the use of NRT products and patients’ reported oral symptoms. This study showed a statistically significantly higher incidence of oral symptoms in current and former NRT users. The reported oral side effects and compounding risk profiles show an imperative need for further research into nicotine replacement therapy products’ impact on oral health status and treatment outcomes in dental patients using NRT.

Tobacco product use is one of the nation’s largest health problems, due to it being the leading cause of drug-related death and hospitalisation.^36^ It is widely recognised that there is no safe way to use tobacco and it is imperative to avoid or stop smoking to prevent fatal conditions.^43^ The paradigm for cessation has changed over the years with a shift towards viewing addiction as a disease and the contemporary approach towards management of tobacco addiction centres around a combination of education, counselling and pharmacotherapy.^20^ A novel method of aiding users in achieving cessation is via remedial substitution of pure nicotine through a tobacco-less product, which has evolved in the form of nicotine replacement products (NRPs).^13,16^

NRPs are the collective name for a range of over-the-counter medications containing pure nicotine. NRPs have been available on the market for the last few decades. The use of NRPs to aid cessation of tobacco is termed nicotine replacement therapy (NRT) and in the contemporary setting is considered the first line of therapy for smoking cessation.^16,29,45,47^

NRT use has been shown to be effective in achieving sustained abstinence, with meta-analysis showing a 50–70% higher chance of quitting.^17,28,45^ Nicotine is the most pharmacologically active component in tobacco, and has a range of effects on the body, including effects on the vascular system, the immune system and the body’s healing mechanisms.^22,26,38^ Of specific interest to oral health professionals are how these effects impact the oral health status. A study in 1999 reported transient mucosal changes following the use of sublingual NRT tablets.^51^ A more recent meta-analysis (2010) showed that the odds ratio of reporting throat soreness and mouth ulcers was statistically significant following the use of NRT products.^28^ However, this study did not look at all possible oral symptoms or the frequency of oral symptoms associated with NRT products.

Knowledge of the adverse effects of NRT on oral tissues will enable health professionals to make better informed clinical decisions.^12,28,50^ This study seeks to examine the incidence of oral symptoms with the use of NRT products.

## Materials and Methods

The research was approved by the Griffith University Human Research Ethics Committee (Protocol Number DOH/05/14/HREC). The paper survey-based study was confidential, open to all current and former smokers over the age of 18 attending the Dental Clinic, School of Dentistry, Griffith University, Gold Coast, Australia. All participants were provided with an information sheet detailing the nature of the research and asked to provide written consent.

The study design was used to analyse the relationship between the use of NRT products and specific oral symptoms experienced by current and former smokers, via a paper-based survey to be completed by the participants.

The survey questionnaires recorded demographic characteristics (age, gender), smoking history (number of years, frequency etc.), NRT history (type of NRT used and number of months or years) and 13 specific oral and systemic symptoms. The sample group was broken into three groups: those quitting with no history of NRT use, former users of NRT, and current users of NRT.

The different types of oral symptoms experienced were recorded to determine which were most commonly associated with NRT. Experience of specific oral symptoms was a dichotomous variable (‘Yes, I experience this symptom’ or ‘No, I do not’), as was NRT use status (Yes/No). Also included for reporting in the questionnaire were several systemic symptoms associated with use of NRPs.

Data were collated, coded, entered and analysed by SPSS Statistics software (Version 22, IBM, Armonk, NY, USA). Demographic data, smoking and NRT history of participants were analysed for frequencies and presented as percentages. The data was analysed using chi-square, Kruskal-Wallis one-way analysis of variance (ANOVA) and Mann-Whitney U test.

## Results

A total of 226 participants completed the survey. The demographic of the participants is shown in Table 1. Smoking status (Table 2) of participants were also recorded. Five different NRT products were reported to be used by the participants (Table 3), with most participants using the products for a short time period of less than 6 months (Table 4). It was observed that the participants used one or more products NRT products and it was hence not possible to assess effects of individual NRT products. However, Table 5 shows reported symptoms of participants who were current smokers and current NRT users versus non-smokers and current NRT users. The data showed no statistical significance.

**Table 1:** Demographic of study sample

N = 226	(%)
**Sex**	
Male	(52.7)
Female	(47.3)
**Age group**	
<20 years	(4.4)
21–30 years	(21.8)
31–40 years	(19.6)
41–50 years	(21.2)
51–60 years	(12.0)
60+ years	(21.3)

**Table 2:** Smoking status

N = 226	(%)
**Years smoking**	
<5 years	(18.2)
5–10 years	(27.3)
11–20 years	(29.5)
21–30 years	(9.1)
30+ years	(15.9)
**Cigarettes per day**	
5 or less	(13.7)
6–10	(31.5)
11–20	(32.4)
21–30	(17.2)
31–40	(2.3)
41–50	(1.8)
50+ a day	(1.4)
**Smoking status**	
Current smoker	(37.6)
Former smoker	(46.9)
Quitting with no NRT product	(3.9)
Quitting with NRT (still smoking)	(5.75)
Quitting with NRT (not smoking)	(5.75)

**Table 3:** Type of NRT used

(For n = 77 previous and/or current users of NRT)	(%)
**Type of NRT used**	
Patch	(55.8)
Chewing gum	(55.8)
Mint or lozenge	(16.9)
Inhaler	(7.8)
Spray	(2.6)
E-cigarette (with nicotine)	(29.9)

**Table 4:** NRT use history

	(%)
**Length of time using NRT**	
<6 months	(64.6)
6–12 months	(19.8)
1–1.5 years	(9.4)
1.5–2 years	(4.2)
>2 years	(2.1)

**Table 5:** Adverse effects analysis: current smokers and users of NRT vs former smokers and current NRT users

Symptom experienced	Current smokers and current NRT product users % (N = 15)	Former smokers and current NRT product users % (N = 17)
Dry mouth	73.3	82.4
Oral ulceration	26.7	17.6
Sore throat	46.7	58.8
Paraesthesia	33.3	41.2
Mucosal irritation	80	64.7

Participants reported experiencing a number of specific oral and systemic symptoms. The most common oral symptom was dry mouth for all three study groups (Table 6). Insomnia was the most commonly reported systemic symptom for the group of smokers currently using NRT (Table 6). Dry mouth, oral ulceration, throat soreness or pain and intraoral parasthesias were significantly more common symptoms in NRT user groups when compared to non-NRT users.

**Table 6:** Bivariate analysis of NRT history: non-users compared to current users of NRT to former NRT users

Symptom experienced	No history of use NRT % (n) (N = 145)	Currently use NRT % (n) (N = 35)	Former user NRT % (n) (N = 43)	Statistical significance
Dry mouth	20.6 (30)	80.0 (28)	65.1 (28)	A>B***≤C NS A>C***
Oral ulceration	13.1 (19)	28.6 (10)	23.3 (10)	A>B **≤C NS A>C***
Throat soreness or pain	11.0 (16)	20 (7.0)	48.8 (21)	A>B***≤C NS A>C***
Intraoral paraesthesia	0.7 (1)	40.0 (14)	18.6 (8)	A>B***>C**
Lip or face paraesthesia	0	17.1 (6)	4.7 (2)	NS
Mucosal irritation	9.0 (13)	60.0 (21)	19.3 (14)	A>B***>C** A>C***
TMJ symptoms	14.5 (21)	28.6 (10)	23.3 (10)	A>B***≤C NS A>C***
Indigestion or stomach upset	13.8 (20)	34.3 (12)	39.5 (17)	A>B***≤C NS A>C***
Gastric reflux	17.2 (25)	28.6 (10)	34.9 (15)	NS A>C**
Regular headaches	15.9 (23)	25.7 (9)	32.6 (14)	NS A>C**
Insomnia	13.1 (19)	60.0 (21)	46.5 (20)	A>B***≤C NS A>C***
Heart palpitations	6.2 (9)	22.9 (8)	16.3 (7)	A>C**
Chest pain	9.7 (14)	8.6 (3)	18.6 (8)	NS

N = total number of participants; n = affected number of participants (n/N*100); Ns = not statistically significant, *p = < 0.05, ** p = 0.01, *** p = 0.001.

## Discussion

In this study, 226 valid cases were included for analysis. The incidence of oral symptoms experienced in current and former smokers quitting with NRT was assessed, and the difference in symptoms between users and non-users of NRT was examined. No other known study has considered specific oral and systemic symptoms in relation to the use of NRT products comparing users and non-users of NRT while quitting or has examined the difference in incidence of specific symptoms experienced in a sample group of both current and former smokers. Studies into NRPs have examined adverse event profiles of NRT but have not considered these effects on oral health status, nor the impact of specific NRT products on the oral environment in detail.^50^ Nicotine toxicity as a result of oral NRT product overusage has recently been recorded; however, that paper’s conclusions were largely based on the overdose of nicotine from NRT and the effects that this produced.^42^ Consideration of the specific oral and dental effects of long-term therapeutic dosing levels of nicotine from NRT has yet to be explored.

This study found that those with a history of NRT use had a significantly higher incidence of dry mouth reported compared to non-users in the participant group (p < 0.001). In this study, a statistically significant proportion of current NRT users reported NRT associated symptoms – especially dry mouth (80%) and mucosal irritation (60%). This present study is supported by the literature that has detailed the adverse effect of the NRT use. The serious side effects of NRT in relation to the oral health environment revealed from this study including dry mouth, oral ulceration, throat soreness or pain, mucosal irritation and intraoral paraesthesia, may be due to the effects of nicotine as a vasoconstrictor, its effects on vascularity of the oral tissues, constant irritation and impaired healing of the oral mucosa.^8,23^

Xerostomia or dry mouth has been noted to be an important risk factor for oral and dental disease^1,35,40,46^ and therefore NRT, with its high-potential for producing dry mouth, must be considered as a risk factor in the management of patients in a dental setting. Furthermore, recent research into nicotine inclusion in toothpaste reported its deleterious effects on the dental tissues and saliva, further supporting the findings of this study.^3^ Modification of the quality and quantity of saliva by NRT may result in an increase in the acidity of the oral environment due to an impairment of the buffering capacity of the saliva. This has ramifications in addition to affecting a patient’s caries-risk profile, and also places denture wearing patients at risk of developing conditions such as candidiasis, impaired taste and speech, mouth ulcerations, soreness, oral mucositis, and dryness and cracking of the vestibular tissue.^2,43,44,46^ This side effect of NRT is highly statistically significant for dentists and oral health professionals, owing to its nature as a modifiable factor in caries-risk management and xerostomia management of patients.

### Oral Ulceration

Current users of NRT were found to be significantly more likely than non-users to experience oral ulceration, with 28.6% (p = 0.025) reporting to have experienced this at some stage as compared to non-users of NRT. The result may be contributed to the occurrence of oral ulceration and aphthous stomatitis as a complication of quitting smoking, which is well documented.^27,49^ The meta-analysis of over 120 studies concluded that mouth ulceration was related to use of NRT,^28^ which supports the present finding of this study. One study into the frequency of aphthous ulceration in quitting smokers noted that users of NRT experienced a lower rate of oral ulceration, in contrast to the findings of this study.^25^

Patients quitting smoking and presenting with oral ulceration should be reassured that this is a common occurrence in the first few weeks of cessation, but should be monitored during the quitting phase for traumatic ulceration and secondary infection, while providing strategies for appropriate management. In order to prevent relapse due to avoidance of therapy, it may be appropriate to recommend a change in mode of NRT for these patients. The results of this study and further research is needed into the role of NRT on aphthous stomatitis.

### Throat Soreness or Pain

Throat soreness or pain was reported by 48.8% of patient with a history of NRT use, compared to just 11% of those with no history of NRT use and current users of NRT. Considering the fact that the study sample was composed of current and former smokers, this finding is particularly interesting. Irritation of the throat has been described as a common adverse effect associated with NRT product use and the results of this study support the existing literature on the subject.^28^ It remains unclear at this stage what impact continued irritation due to chronic NRT use, and the relative risk in the development of oral mucosal changes is. Nicotine’s immunosuppressive effects^14,15,21,26,32,39^ and their role in dry mouth may also contribute to a higher experience of sore throat as a result of common infections experienced by NRT users; however, this is an area requiring further investigation.

### Intraoral Paraesthesia

Almost 40% of current users NRT products reported experiencing intraoral paraesthesia compared to 0.7% of non-users (p < 0.001). These results suggest that there is a statistically significant proportion of NRT users who experience this symptom directly as a result of NRT use, which supports the findings of a Cochrane Review of 120 studies on side effects on NRT^28^; indeed, this is a very commonly reported symptom from users of NRT and may be related to the high topical concentrations of nicotine in the mouth. The capacity for nicotine to penetrate to a deep level of tissue highlights the pathogenic potential for its effects on the oral mucosa. The extent of NRT’s capacity to elicit burning mouth syndrome and other irritant-stimulated oral neuralgias and paraesthesia remains unexplored. Clinicians and patients alike should be made aware of NRT’s ability to elicit such reactions to aid accurate diagnosis and appropriate management.

### Mucosal Irritation

Current users (60%) reported mucosal irritation, compared with 9% of those of non-users of NRT before, representing a statistically significant difference in incidence for this symptom (p < 0.001). Studies have also highlighted nicotine’s potential to elicit oral lichen planus, lichenoid reactions, contact stomatitis, epithelial dysplasia, oral submucous fibrosis, erythema bullous reactions, leukoplakia and premalignant changes.^7,9,19,48^ The ability for NRPs to elicit mucosal irritation may have an impact on patients’ post-surgical and periodontal healing progression.

Compromised immune function^14,15,21,26,33,39^ and constant localised irritation of the mucosa through nicotine exposure predispose the patient to secondary infection and may impair the healing of soft and hard tissue, exacerbating nicotine dependent potential tissue transformation.^10,18,24,30,31,37,41^ This may have prospective implications for surgical, regenerative, grafting, mucogingival, periodontal and dental-implant procedures intraorally, as well as in the general development of other oral soft tissue pathology. Nicotine’s potential role as a factor in developing osteonecrosis and tissue modifying capability should be further investigated.

### Systemic Symptoms

Compared with non-users, several systemic symptoms in current and former users were reported. Statistically significant symptoms included indigestion or stomach-upset (p < 0.001), regular headaches, insomnia (p < 0.001) and heart palpitations. Gastrointestinal symptoms, due to accidental ingestion of nicotine by swallowing nicotine saturated saliva from oral NRT products has been reported as a potential cofactor for gastric cancers.^52^ Promotion of nausea or vomiting and reflux has implications for erosive damage of the dentition due to stomach acids being present in the oral environment. Sleep disturbance, headaches leading to temporomandibular joint (TMJ) symptoms and cardiac symptoms may be related to nicotine’s stimulatory effect on the sympathetic nervous system.^4,22^

Current users of NRT experienced a significantly higher number of symptoms compared to those never having used NRT previously (p < 0.001), and a similar statistically significant difference was found between previous users of NRT and non-users (p < 0.001). There was no statistically significant difference between current and previous users of NRT for a number of symptoms, suggesting that the effects of NRT may persist beyond the period of immediate NRT use. However, as this study was limited in its ability to determine the length of time passing between cessation of NRT use and the recording of symptoms, a definitive conclusion is difficult to draw at this stage.

A full history of a patient’s experience of oral symptoms and other factors should be considered prior to recommending NRT. Dentists should be equipped to provide patients with accurate advice and counsel regarding NRT use. The recommendation of products for smoking cessation and the use of NRT should be undertaken with particular focus on an individual’s risk profile, with consideration of modifying factors associated with NRT use and expected treatment outcome. At present, there are is a lack of available resources for guiding the general dental practitioner and an absence of readily available guidelines for counselling the patient in smoking cessation products, and the consequences of these products on dental, post-surgical or periodontal management.^11^

That smoking is a major risk factor in exacerbating pathological progression, and destruction of periodontal tissue prejudices the management of periodontal conditions and post-surgical healing, is well established.^5,6,33,34^ Smoking cessation is imperative to optimise clinical outcomes and to promote appropriate clinical decision-making, however, consideration should also be given to NRT and its deleterious side effects which may modify response to treatment. Overall sequel may include a lack of patient compliance in order to avoid the NRT-induced adverse effects, and thereby relapse into smoking habits may occur. Hence, further evaluation in this field is required for optimal control of smoking and adjunct usage of products to achieve a beneficial outcome.

### Limitations

The socioeconomic and age group demographics of the patient base at the University Dental Clinic may differ from the wider population. Other potential compounding variables such as age, sex, smoking history, mode of NRT use and the inability to define a true causal relationship between the independent and dependent variables may be considered as a limitation of this study. The study did not look at the magnitude, severity and frequency of reported symptoms. Future research should focus on identifying and controlling such compounding variables and be given due consideration when performing analysis.

## Conclusions

Dental professionals do not routinely consider the effect of NRT in the oral and dental management of patients. Dental professionals should be well versed with the potential side effects of NRT products and be competent in the management of oral symptoms. It could be assumed that appropriate management of NRT-related side effects may increase compliance and better smoking cessation outcomes for the patient.
